# Root coverage with apical tunnel approach using propolis as a root conditioning agent: A case report with 2‐year follow‐up and review of the literature

**DOI:** 10.1002/cre2.751

**Published:** 2023-06-20

**Authors:** Obada Mandil, Hamoun Sabri, Neshatafarin Manouchehri, Diana Mostafa, Hom‐Lay Wang

**Affiliations:** ^1^ Department of Periodontics and Oral Medicine, School of Dentistry University of Michigan Ann Arbor Michigan USA; ^2^ Center for Clinical Research and Evidence Synthesis In Oral Tissue Regeneration (CRITERION) Ann Arbor Michigan USA; ^3^ Department of Periodontics and Oral Medicine, School of Dentistry Alexandria University Alexandria Egypt

**Keywords:** apical tunnel approach, root coverage, gingival recession, propolis

## Abstract

**Objectives:**

One of the main challenges in performing root coverage is patient discomfort and donor site morbidity. This case report presents a minimally invasive apical tunnel surgical technique, with propolis for root conditioning, to correct gingival recession defects without harvesting donor grafts, flap elevation, or sutures. Propolis is a natural anti‐infective, anti‐inflammatory, and antioxidant agent.

**Material and Methods:**

A 58‐year‐old woman with no significant medical history was presented for root coverage of her upper left canine and first premolar with recession type (RT)1A (+). Propolis was used as a root conditioning agent to promote soft tissue coverage via an apical tunnel approach. During the apical tunnel approach, a small apical hole was made 6 mm below the mucogingival junction, and the mucosa and associated attached gingiva was away from the tooth so the flap could be repositioned coronally. Collagen matrix was used as a soft tissue graft material.

**Results:**

At the 2‐month, 6‐month, 8‐month, and 2‐year follow‐up, complete root coverage was achieved for both teeth. No bleeding on probing was noticed nor recurrent GRs at the treated sites.

**Conclusion:**

Without incisions, donor site reflection, or flaps, the apical tunnel approach can be successfully used to cover the exposed roots. Additionally, propolis is a potential root conditioning agent during soft tissue graft procedure due to its anti‐inflammatory and antioxidant properties.

## 1 INTRODUCTION

Periodontal plastic surgery is performed to address mucogingival defects. The apical movement of the gingival margin brought on by various diseases or illnesses and linked to clinical attachment loss is now known as gingival recessions (GRs) according to the 2018 World Workshop classification (Romandini et al., 2020). Numerous surgical techniques have been employed to correct GRs (Stefanini et al., 2023). Despite the large number of research that has been conducted on this topic, the main challenges continue to be surgical considerations, time constraints, and patient discomfort. According to the American Academy of Periodontology, the mean root coverage of GRs varies from 67% to 86%, depending on the surgical approach and prognosis of the GR defect. Also, the position of the tooth within the arch, the thickness of the gingival biotype, and the existence or absence of keratinized tissue are considered challenging factors (Javaid et al., 2020).

Apical tunnel surgical (ATS) techniques, such as the pinhole approach, are a minimally invasive surgical procedure that reverses GRs without the need for donor grafts, flap elevation, or sutures (Beck, 2018; Chao, 2012). The alveolar mucosal tissue is punctured with a needle. Through this tiny opening, specialized tools are used to gently release the gingival tissues and move the gingiva to cover the exposed root surface. By doing this, all muscle and fibrous adhesions are broken up, allowing the flap to move freely and tension‐free in the coronal direction (Chao, 2012).

Several biologics have been proposed to be used for root conditioning agents during the root coverage procedures (Ahangari et al., 2018). Propolis refers to a broad range of resinous materials that honeybees gather from plant parts, buds, and exudates (Martinotti & Ranzato, 2015). Propolis has been used in dentistry (Ahangari et al., 2018) due to its ability to condition the roots and to promote faster healing, as well as to act as an anti‐infective, anti‐inflammatory, and antioxidative agent (Velazquez et al., 2007). It has been demonstrated that propolis is efficient against oral bacteria, pointing to its potential as an anti‐periodontal pathogen, including Porphyromonas gingivalis, as well as an anti‐cariogenic agent (Coutinho, 2012; Fard, 2022). Also, Galangin, genistein, quercetin, resveratrol, caffeic acid, and caffeic phenyl ester are active substances found in propolis that enhance its antiseptic, antibacterial, antifungal, antiviral, and immunomodulatory properties (Martinotti & Ranzato, 2015). Literature has also shown propolis is able to trigger an increase in extracellular matrix components during early wound repair thus lead to stimulation of the transforming growth factor‐ß (TGF‐ß) expression in promoting hemostasis and control inflammation (Martinotti & Ranzato, 2015).

Despite its feasibility, the ATS still lacks evidence regarding its long‐term outcomes. Therefore, the aim of this case‐report was to present a GR case that was successfully managed with ATS along with application of propolis to conditioning the root surface. Additionally, a narrative literature review was aimed to be performed to map the current evidence success, and remarks on the use of this surgical technique.

## 2 LITERATURE REVIEW

In this report, to provide a brief history and evidence on the use of AST, we also performed a literature review. To do so, PubMed database was searched. The Keywords consisted of (ATS OR apical tunnel approach OR pinhole technique) AND (Root AND coverage). No language and date limitations were applied. The search was performed in December 2022.

The results of our literature search revealed 16 articles of which eight articles were reviewed. Four of them were case series and the other four were case reports. These articles were published between 2012 and 2021. Two articles used ATS with platelet rich‐fibrin (PRF) (Anuroopa, 2018; Nibudey & Baliga, 2020) as an adjunctive agent, while one article used titanium‐prepared platelet rich‐fibrin (T‐PRF) (Agarwal et al., 2020). Five articles used only ATS with collagen matrix. Also, their follow‐up period ranged from 3 to 18 months. Regarding the results of the reviewed studies, in five of them, the authors achieved complete root coverage and a gain in keratinized tissue was reported in four of them. Table 1 depicts the summary of the clinical outcomes as well as the techniques that were employed in the reviewed articles.

**Table 1:** The characteristics of the included studies in the literature review. The results of this review revealed a lack of sufficient evidence on the long‐term outcomes of the apical tunnel surgical (ATS) technique.

Reference	Subjects	Study design	Last follow up	Outcomes
Chao (2012)	43 patients	Case series	18 months	**Root coverage:** 88.4% **KT gain:** 1.3 mm. **PD reduction:** 1.4 mm. **CAL gain:** 4.4 mm.
Reddy et al. (2017)	5 patients	Case series	6 months	**Root coverage:** 96.7% complete root coverage, while two sites had partial coverage. **Mean WKT:** preoperatively as 2.11 mm and postoperative as 2.78 mm.
Anuroopa et al. (2018)^a^	2 patients	Case report	6 months	**Root coverage:** 93%–95% with complete coverage. **KT gain:** Mean of 1.2 mm.
Sharma et al. (2019)	2 patients	Case report	6 months	**Root coverage:** 100%
Agarwal et al. (2020)	10 patients	Case series	6 months	**Root coverage:** 98% at 3 months follow‐up, 87% at 6 months follow‐up. **KT gain:** Significant increase from baseline to 6 months follow‐up.
Nibudey and Baliga (2020)^a^	22 patients	Case series	6 months	Qualitatively reported improved clinical outcomes
Mostafa and Mandil (2021)	1 patient	Case report	8 months	**Root coverage:** 100%
Chandramohan et al. (2021)	1 patient	Case report (Split Mouth, Vs CAF)	3 months	**Root coverage:** 100% **KT gain:** 2 mm. **PD reduction:** 50% **CAL gain:** 3 mm.

*Note*: Also, there is no study reporting the long‐term outcomes of this method, as of the 2‐year follow‐up in this study.

Abbreviations: CAF, coronally advanced flap; CAL, clinical attachment level; KT, keratinized tissue; PD, pocket depth; WKT, width of keratinized tissue.

^a^ This study used a modification to the original technique by addition of sutures.

## 3 CASE PRESENTATION

This report is in accordance with the Declaration of Helsinki of 1975, as revised in Fortaleza in 2013 and the manuscript has been prepared according to the CARE guidelines for improving research reporting in case reports (Supporting Information).

A 58‐year‐old female patient was referred with the main complaint of receding gums and visible roots of teeth #11 and #12 (ADA numbering system). The past medical history was clear without history of any drug consumption.

### 3.1 Clinical and periodontal examination

**Figure 1:**
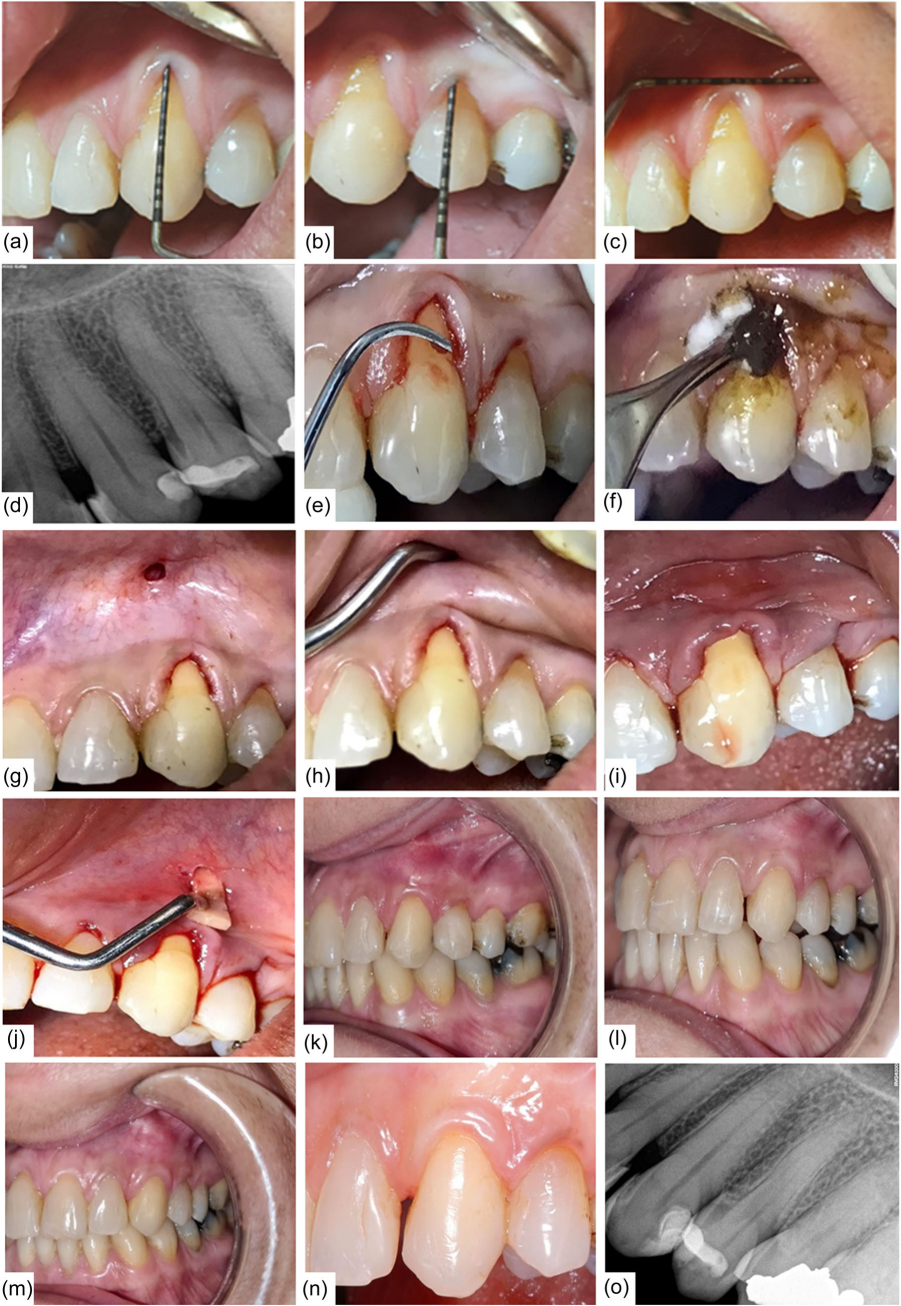
Probing depth (PD) was 1 mm in the midfacial region for both teeth without any bleeding on probing (a, b). The recession height was 4 mm in tooth #11 (a) and 2 mm in tooth #12 (b), and the width of keratinized tissue (WKT) was 2 mm for both teeth (a–c). A preoperative periapical radiograph was taken, the affected teeth showed no signs of interproximal radiographic bone loss as well as no interproximal attachment loss (d). Only a slight amount of gingival inflammation was seen, along with rolled marginal gingiva and blunted interdental papillae. First, scaling and root planing was performed to prepare the teeth for phase II treatment (e). Local anesthetic infiltration was performed and applied propolis paste using a cotton pellet to condition the roots for 3 min (f). Then pierced an apical hole of 4 mm above the mucogingival junction (MGJ) apical to the recession defect (g). The gingiva then pushed downward until it is coronal to cementoenamel junction, using a curved Orban's knife and tunneling tools to loosen gingival tissues. Full‐thickness splitting was accomplished, resulting in free mobile gingiva (h, i). To help and hold the gingiva in its coronal position, one‐piece of CenoMembrane, an allograft collagen matrix (size: 10 × 10 mm, thickness: 0.6–0.9 mm, CenoBiologics Ltd–Tissue Engineering, United Kingdom) was divided into small strips and placed through the hole to the interdental papillae, as illustrated in (j). To keep the gingiva in its new position, gentle digital pressure was applied for 3 min. Propolis was then reapplied to the gingiva for an additional 3 min. The 2 months (k), 6 months (l), 8 months (m), and 2 years (n) follow‐ups showing complete root coverage. The pocket depth was 2 mm for both teeth #11 and #12 during all follow‐up visits (k–n). WKT of both affected teeth increased to 3 mm and remained the same during all follow‐ups (k–n). Post‐op peri‐apical X‐ray taken at the 2 years follow‐up showed no bone level change when compared to baseline (o).

Initially, periodontal examination was performed. (Figure 1) presents the baseline clinical findings. The average plaque index was 0.9. The patient expressed that she manually cleaned her teeth using a horizontal motion brushing technique, twice daily without any auxiliary tools.

### 3.2 Treatment considerations and surgical procedure

According to AAP World Workshop classification, teeth #11 & #12 were classified as RT1 A (+) (Cortellini & Bissada, 2018). The patient declined to receive an autogenous soft tissue graft. Thus, the ATS technique was indicated for her to cover the exposed root surfaces. The phase I treatment was performed before the surgical phase. The ATS was employed by utilizing propolis as a root conditioning agent to enhance clinical outcomes and healing. Figure 1e–j depicts the surgical steps. After the surgery, the post‐op instructions were given, additionally, she was asked to apply a propolis paste twice daily for the first week. After 10 days following the surgery, chlorohexidine mouthwash was prescribed to avoid any physical disruption. After 4 weeks, the patient was told to brush in vertical strokes.

### 3.3 Clinical outcomes

At the 2‐month (Figure 1k), 6‐month (Figure 1l), 8‐month (Figure 1m), and 2‐year (Figure 1n) follow‐up, complete root coverage was achieved for both teeth. No bleeding on probing was noticed nor recurrent GRs at the treated sites. The patient expressed satisfaction with the esthetic results that were obtained. Figure 1o shows stable bone level at 2‐year follow‐up.

## 4 DISCUSSION

In this report, we demonstrated that a successful root coverage was achieved and maintained for 2‐year after ATS. In this case, propolis was used as a root conditioning agent. To the best of our knowledge, this is one of the first case reports that used this combination to manage the GR defect.

The advantages of this minimally invasive surgical procedure comprise coverage of the exposed roots without the need for an incision, a donor site, with providing rapid esthetic outcomes, and taking less chair‐time, and covering multiple GRs. Propolis has been utilized in several other periodontal procedures with improved clinical outcomes (enhanced wound healing and reduced inflammation) (Filho & Carvalho, 1990; Nakao et al., 2020; Zohery et al., 2018). Hence, it is our intent to apply propolis to condition the exposed root surface because it is a natural anti‐infective and antioxidant agent so it can minimize inflammation as well as speed up the healing process. Our observations confirm this finding, since no surgical bleeding or inflammation was observed. This may be related to the anti‐inflammatory properties of propolis, which inhibits lipoxygenase and cyclooxygenase enzymes, prevention of the conversion of arachidonic acid to prostaglandins and leukotrienes (Kuropatnicki et al., 2013), as well as the stimulation of expression of TGF‐ß (Martinotti & Ranzato, 2015), that aids in hemostasis during wound healing.

Due to the minimally invasive nature of this technique, without separation of underlying tissue, less bleeding, improved access and visibility, and surgical time was experienced. This also explains why we did not note any complications during healing nor the formation of scars, which has further confirmed the biological and esthetic benefits of ATS approach (Anuroopa, 2018).

In both teeth #11 & #12, our findings showed an increase in gingival volume and thickness. These findings also supported others, who concluded that ATS could increase tissue volume and produce predictive results if the baseline tissue thickness is at least 0.8–1 mm. Additionally, there was an improvement in thickness and width of keratinized tissue (WKT) in both teeth from the baseline to 8 months and remained stable at 2 years. This observation agreed with many of the previous findings (Anuroopa, 2018; Kuropatnicki et al., 2013; Nakao et al., 2020; Stefanini et al., 2023). Also, the rationale for using the collagen matrix in this technique was to maintain the level of gingival margin (Barootchi et al., 2022), as well as to modify the gingival phenotype (Barootchi et al., 2020). Moreover, collagen matrix is a noncross‐linked, resorbable, and intended to promote angiogenesis and tissue integration (Barootchi et al., 2020), which all agree with our long‐term results.

Nonetheless, the ATS also has some challenges such as technique sensitivity and the need for specialized instruments to elevate the flap without exposing the inner tissue, which raises the risk of flap proliferation. In addition to these, there is still a lack of quality research on the efficacy of this technique being supported by randomized controlled trials compared to other techniques. Therefore, despite the long‐term feasibility of ATS that is shown in this case, the readers should be cautious about the limitations of the case‐report nature of this study. Lastly, the results of the literature review regarding implementing ATS in root coverage procedures indicated a substantial gap in the higher level of evidence articles. Thus, it is suggested for future research to experiment on this technique and its feasibility within controlled trials with larger sample sizes.

## 5 SUMMARY

The ATS technique is a method that shows promising results for treating GRs. The use of propolis paste for this technique should be taken into consideration for root cleansing and improved healing. However, future randomized controlled trials are needed to validate the current findings.

## AUTHOR CONTRIBUTIONS

None.

## CONFLICT OF INTEREST STATEMENT

The authors declare no conflict of interest.

## Supporting information

Supporting information.

## Data Availability

All data regarding this article will be provided upon request from the authors. The corresponding author can provide the data that were utilized to support the study's conclusions upon request.
